# Melatonin Ameliorates Thermotolerance in Soybean Seedling through Balancing Redox Homeostasis and Modulating Antioxidant Defense, Phytohormones and Polyamines Biosynthesis

**DOI:** 10.3390/molecules26175116

**Published:** 2021-08-24

**Authors:** Muhammad Imran, Muhammad Aaqil Khan, Raheem Shahzad, Saqib Bilal, Murtaza Khan, Byung-Wook Yun, Abdul Latif Khan, In-Jung Lee

**Affiliations:** 1 School of Applied Biosciences, Kyungpook National University, Daegu 41566, Korea; m.imran02@yahoo.com (M.I.); aqil_bacha@yahoo.com (M.A.K.); murtazakhan.bio@gmail.com (M.K.); bwyun@knu.ac.kr (B.-W.Y.); 2 Department of Horticulture, University of Haripur, Haripur 22620, Pakistan; raheemshehzad@ymail.com; 3 Natural and Medical Sciences Research Center, University of Nizwa, Nizwa 616, Oman; saqib@unizwa.edu.om; 4 Department of Engineering Technology, College of Technology, University of Houston, TX 77479, USA

**Keywords:** melatonin, polyamine, antioxidant, abscisic acid, salicylic acid, soybean, heat tolerance

## Abstract

Global warming is impacting the growth and development of economically important but sensitive crops, such as soybean (Glycine max L.). Using pleiotropic signaling molecules, melatonin can relieve the negative effects of high temperature by enhancing plant growth and development as well as modulating the defense system against abiotic stresses. However, less is known about how melatonin regulates the phytohormones and polyamines during heat stress. Our results showed that high temperature significantly increased ROS and decreased photosynthesis efficiency in soybean plants. Conversely, pretreatment with melatonin increased plant growth and photosynthetic pigments (chl a and chl b) and reduced oxidative stress via scavenging hydrogen peroxide and superoxide and reducing the MDA and electrolyte leakage contents. The inherent stress defense responses were further strengthened by the enhanced activities of antioxidants and upregulation of the expression of ascorbate–glutathione cycle genes. Melatonin mitigates heat stress by increasing several biochemicals (phenolics, flavonoids, and proline), as well as the endogenous melatonin and polyamines (spermine, spermidine, and putrescine). Furthermore, the positive effects of melatonin treatment also correlated with a reduced abscisic acid content, down-regulation of the gmNCED3, and up-regulation of catabolic genes (CYP707A1 and CYP707A2) during heat stress. Contrarily, an increase in salicylic acid and up-regulated expression of the defense-related gene PAL2 were revealed. In addition, melatonin induced the expression of heat shock protein 90 (gmHsp90) and heat shock transcription factor (gmHsfA2), suggesting promotion of ROS detoxification via the hydrogen peroxide-mediated signaling pathway. In conclusion, exogenous melatonin improves the thermotolerance of soybean plants and enhances plant growth and development by activating antioxidant defense mechanisms, interacting with plant hormones, and reprogramming the biochemical metabolism.

## 1. Introduction

Various environmental stresses, such as temperature, drought, heavy metals, and salinity, are a part of climate change and pose a great threat to crops. Among abiotic stresses, heat stress is among the most detrimental to crop growth and development [1]. A 2018 Intergovernmental Panel on Climate Change (IPCC) report stated that crop yields would experience severe and widespread impacts if global warming exceeds 1.5 °C above pre-industrial levels [2]. As of 2017, the average global warming increase was 1 °C above pre-industrial levels. Climate change will continue in the decades ahead, with the world already committed to a temperature increase in the next two decades due to the 20–30-year lag in the global climate system. In countries such as Asia, Africa, and the Middle East, a 3–4 °C increase in temperature could decrease crop productivity by up to 35% [3].

Temperature alters all aspects of plant processes, including growth, development, physiological processes, and yield. One major consequence of high-temperature stress is oxidative damage caused by the excess accumulation of hydrogen peroxide (H_2_O_2_), superoxide anion radical (O_2•_^−^), and hydroxyl radical (OH•) [4], resulting in excess production of malondialdehyde (MDA), reduced membrane permeability, stability, and mobility and enzyme denaturation [1,4]. To protect against heat-induced oxidative damage, plants have developed an antioxidant defense system for the detoxification of reactive oxygen species [5] that involves several enzymatic and non-enzymatic antioxidants, such as catalase [6], peroxidase (POD), ascorbate peroxidase (APX), glutathione reductase, glutathione [7], superoxide dismutase (SOD), and dehydroascorbate [8], besides carotenoids and phenols [9]. Moreover, heat shock proteins (HSPs) and heat shock transcription factors (HSFs), such as HsfA2, play an important role in plant stress tolerance by regulating the antioxidant defense system and scavenge excess ROS generated when the plant is exposed to stress [10].

Melatonin, as a plant hormone was first discovered in the pineal gland of vertebrates and was later found in at least three clades of bacteria including dinoflagellates, multicellular and unicellular fungi, and approximately 120 plant species. In plants, its concentrations are widely dependent on species and environmental factors. In plants, tryptophan is the first step of melatonin that converts to tryptamine and tryptamine then convert to serotonin through T5H [11]. Similarly, melatonin is converted to 2-hydroxymelatonin through 2-oxoglutarate-dependent dioxygenase (M2H), however, its function in plants still remains to be determined, but its lack in cyanobacteria suggests that it has a plant-specific function. In dinoflagellates, the melatonin is deacetylated to a bioactive metabolite 5-methoxytryptamine, where in plants, dinoflagellates and vertebrates, and another metabolite AFMK can be formed non-enzymatically and enzymatically [12]. Photocatalytic AFMK can form in a significant quantity under visible light and in the presence of the hydroxyl radical scavenger dimethylsulfoxide. Therefore, AFMK is considered a radio-protector in the human epidermis and it may also be in plants [13].

Moreover, in plants, melatonin (MT) has garnered intense research interest due to its protective role against various biotic and abiotic stresses (drought, salinity, heavy metals, oxidative stress, and high temperature) [11,14]. MT modulates the antioxidant enzyme activities to improve ROS (H_2_O_2_, O_2_^−^) detoxification, which is considered a fundamental approach toward the enhancement of stress tolerance [15]. Furthermore, MT enhances the ascorbate-glutathione cycle and reprograms the polyamine (PA) metabolic pathways to scavenge excess ROS, maintain cellular membrane stability, and protect plants from heat-induced oxidative stress [5]. These remarkable properties of MT as a ROS scavenger and antioxidant molecule are widely reported in plants spices [16,17,18,19]. Moreover, MT can induce gene expression under stress conditions by enhancing growth and development during seed germination [18,19]. Tomato [5], Kiwifruit [20], radish [21], melon seedlings [22], and maize [23] are among many plants in which exogenous MT exerts a protective role against induced heat tolerance by modulating gene expression and promoting the antioxidant defense system. Moreover, industrial food wastes can also be a great source of melatonin production, therefore, the use of food wastes can be a more environmentally friendly strategy for plant growth and development. However, the proper segregation of bio-wastes and using appropriate biotransformation approaches can provide high energy recovery and zero pollution [24]. In addition, several studies have demonstrated that MT promotes cellular protein protection through induction of HSPs and autophagy in response to stress [25,26]. Exogenous application of melatonin and its derivatives have a positive impact on preventing UVB-induced oxidative stress and improved the tissue, cellular, and genomic integrity against UVB when exposed to UV [27]. Melatonin also has a potential impact on the reduction of heat shock Hsp70 protein in UVR-treated, full-thickness skin in organ culture and cultured keratinocytes [28]. Additionally, MT application increases the endogenous MT and cytokinin levels, decreases abscisic acid [29] accumulation under heat stress [30], and protects against heat-induced protein oxidation by reducing oxidative stress and decreasing the ratio of insoluble protein to total protein [25].

Soybean is an important food energy source with high economic value, oil quality, protein, and various minerals and vitamins [29]. However, soybean is heat-sensitive. Heat stress affects the growth, development, physiological, and biochemical attributes of plants, causing a decrease in photosynthetic activities, premature leaf senescence, and limited yield during vegetative and reproductive stages [31,32,33]. The effects of heat stress on the plant photosystems reduce the nutrition and water supply, further influencing the internode elongation and leaf expansion and reducing crop growth and yield [29,32].

As mentioned above, MT acts as a positive plant growth regulator and plays a role in heat stress tolerance in many plants species, such as tomato [5], kiwifruit [20], and Arabidopsis [26]. Similarly, in arabidopsis mutant *d14-1* and *max4-1* the endogenous tissue melatonin act downstream of strigolactone to regulate *Flowering Locus C* and thus inducing delay to flowering [34]. However, the role of MT in thermotolerance in soybean has not yet been observed. Therefore, the current study aimed to elucidate the mitigating role of MT against heat stress in soybean plants, highlighting its effective role in ROS (H_2_O_2_, O_2_^−^) homeostasis through the modulation of antioxidant enzyme defense systems, such as SOD, APX, CAT, POD, and GSH, heat stress-response transcription factors (such as HSFA2) and HSPs (such as HSP90), and ABA and its biosynthesis gene NCED (9-cis-epoxycarotenoid dioxygenase). In addition, we also evaluate the possible effects of MT application on the PA accumulation in response to heat stress in soybean plants.

## 2. Results

### 2.1. Exogenous MT Improves Morphological Parameters of Soybean under Heat Stress

**Figure 1:**
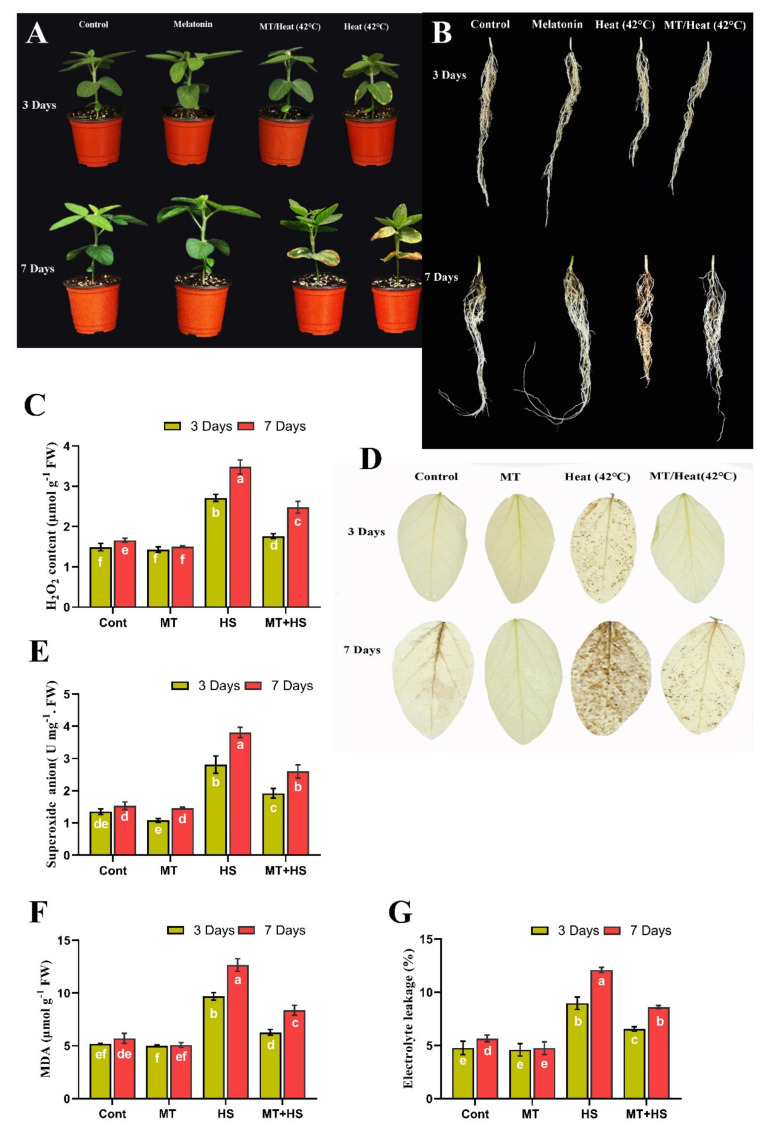
Effects of melatonin application with or without high temperature (42 °C) in soybean seedling after 3 and 7 days, phenotypical visualization of (**A**) shoot (**B**) root, (**C**) hydrogen peroxide, (**D**) DAB staining, (**E**) superoxide anion, (**F**) MDA level, and (**G**) electrolyte leakage. Each data point is the mean of three replicates. Error bars represent the standard error of the mean. Bars with different letters are significantly different from each other by Duncan’s multiple range test at *p* ≤ 0.05.

Several plant growth attributes were measured on days 3 and 7 during heat stress. Soybean SL, RL, biomass, and Chl content were decreased significantly by 16.5%, 14.4%, 33.3%, and 18.5%, respectively, after heat stress for 3 days compared to control plants (Figure 1A and Table 1). However, exogenous MT reversed these negative effects of heat stress, increasing soybean SL, RL, biomass, and Chl content by 15.5%, 20.4%, 35.7%, and 20.5%, respectively, compared to the heat-stressed controls (Table 1). A similar trend was observed on day 7, with a decreases of 17.1% (SL), 21.5% (RL) 37.9% (FW), 45.8% (DW), and 29.7% (Chl content) in the heat-treated plants compared to control plants (non-treated), while MT-treated plants significantly increased these parameters by 19.8% (SL), 16.5% (RL), 33.3% (FW), 46.1% (DW), and 22.5% (Chl content) compared to control plants (42 °C treated) (Table 1 and Figure 1A,B).

To determine whether exogenous MT could delay the leaf senescence, we quantified the Chl (a and b) and carotenoid contents in soybean leaves on days 3 and 7 during heat stress. Exposure to high temperature for 3 days caused a significant decrease in the Chl a (16.5%), Chl b (23.3%), and carotenoid (64.9%) contents, which were further decreased by 48.6%, 33.1%, and 61.4%, respectively, on day 7 compared to control plants (non-treated) (Supplementary Materials Figure S1). However, MT treatment inhibited the degradation of all three pigments, with significantly higher levels recorded in MT-treated plants on days 3 and 7 (48.2% and 66.1% Chl a, 54.6% and 43.4% Chl b, 83.4% and 87.2% carotenoid) than in heat stress-treated plants (Supplementary Figure S1).

### 2.2. Exogenous MT Scavenges Over-Accumulated ROS, Reduces MDA, and Decreases Electrolyte Leakage Level under Heat Stress

In response to stress stimuli, plants generate ROS, such as O_2_^−^ and H_2_O_2_, which accumulate in the cells, causing oxidative damage and inducing programmed cell death. Therefore, we investigated the effects of exogenous MT on ROS production in soybean plants under heat stress. High temperature (42 °C for 3 and 7 days, respectively) caused a significant increase in O_2_^−^ (87.9% and 98.6%) and H_2_O_2_ (72.2% and 109.6%) levels compared to control plants (non-treated). These effects were reversed by exogenous MT, which significantly decreased the over-accumulation of O_2_^−^ (31.7% and 36.6%) and H_2_O_2_ (35.1% and 29.7%) (Figure 1C–E).

It is well known that an increase in ROS generation may lead to oxidative damage and increase the MDA level and electrolyte leakage. To further investigate the effect of MT treatment, we quantified the MDA level and electrolyte leakage content in the heat-stressed (42 °C) plants. Heat stress significantly increased the MDA level and electrolyte leakage by 61.2% and 72.4% on day 3 and 112.6% and 89.8% on day 7 compared to control plants (non-treated). With MT treatment, these effects of high-temperature stress were significantly reversed; MDA and electrolyte leakage contents decreased by 42.7% and 28.7% on day 3 and 33.8% and 37.6% on day 7, respectively, compared to the heat-stressed control plants (Figure 1F,G).

### 2.3. Exogenous MT Enhances Antioxidant Activity under High Temperature Stress

**Figure 2:**
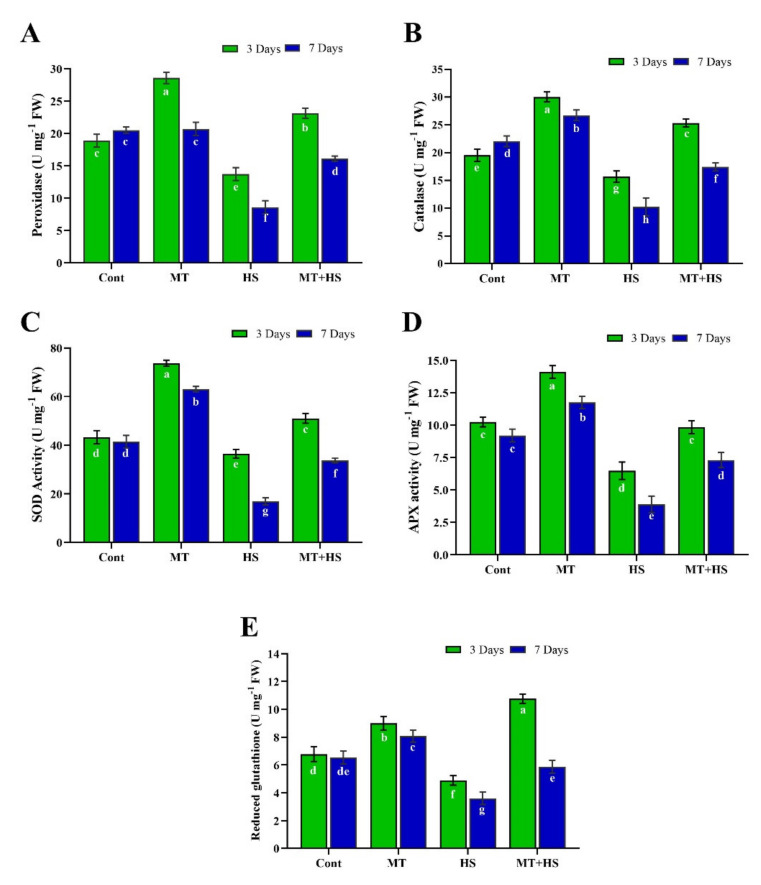
Effects of melatonin application with or without high temperature (42 °C) on antioxidant enzyme activities of (**A**) peroxidase, (**B**) catalase, (**C**) superoxide dismutase (**D**) APX activity, and (**E**) reduced glutathione in soybean seedling after 3 and 7 days. Each data point is the mean of three replicates. Error bars represent the standard error of the mean. Bars with different letters are significantly different from each other by Duncan’s multiple range test at *p* ≤ 0.05.

MT is a well-known antioxidant stimulator that is also involved in plant growth and development and stress tolerance. To validate these effects, we investigated the exogenous application of MT on the activation of antioxidant activity in heat-stressed soybean plants. Data shown in (Figure 2A–D) demonstrated that high temperature (42 °C) for 3 and 7 days, respectively, caused a significant decrease in POD (74.5% and 112.3%), CAT (19.8% and 86.4%), SOD (24.9% and 64.5%), and APX (36.7% and 58.5%) activity compared to control plants (without stress). Exogenous MT significantly reversed these adverse effects of heat stress, enhancing the activity of POD (59.3% and 53.3%), CAT (58.6% and 69.3%), SOD (40.1% and 83.5%), and APX (51.8 and 82.1%) on day 3 and day 7, respectively, compared to heat-stressed plants. Furthermore, although GHS content was enhanced by exogenous MT compared to control plants on days 3 and 7 under the normal condition (no heat stress), heat stress for 3 and 7 days significantly reduced the GSH content by 27.8% and 44.8%, separately, in comparison to control plants (no stress). However, exogenous MT reversed the heat stress-induced decrease in GSH content, enhancing its activity by 51.8% on day 3 and 74.7% on day 7 compared to heat-treated (42 °C) plants (Figure 2E).

**Figure 3:**
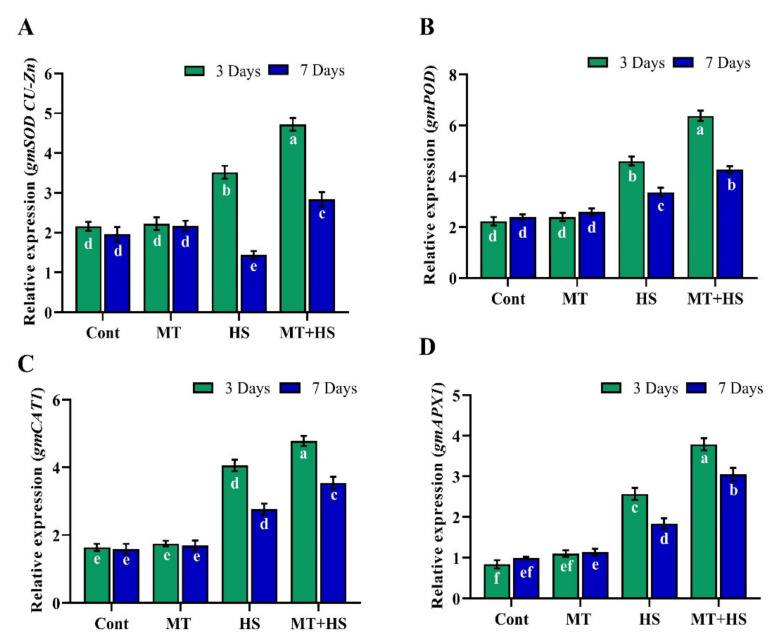
Effects of melatonin application with or without high temperature (42 °C) on the relative expression of (**A**) gmSOD (CU-Zn), (**B**) gmPOD, (**C**) gmCAT, and (**D**) gmAPX in soybean seedling after 3 and 7 days. Each data point is the mean of three replicates. Error bars represent the standard error of the mean. Bars with different letters are significantly different from each other by Duncan’s multiple range test at *p* ≤ 0.05.

To further validate these results, we determined the relative gene expression levels of SOD, POD, CAT, and APX by qRT-PCR analysis. The results in (Figure 3) showed a significant down-regulation in the relative expression of GmSOD1 (25.4% and 49.2%), GmPOD (27.8% and 21.0%), GmCAT (15.6% and 22.0%), and GmAPX (32.1% and 39.6%) after exposure to high temperature (42 °C) for 3 and 7 days compared to MT-treated plants, which revealed significant upregulation in the expression levels of GmSOD1 (41.0% and 83.3%), GmPOD (38.6% and 26.7%), GmCAT (21.1% and 28.2%), and GmAPX (47.4% and 65.7%).

### 2.4. Effects of Exogenous MT on Phenolic and Flavonoid Contents and Antioxidant (DPPH) Activity under Heat Stress

**Figure 4:**
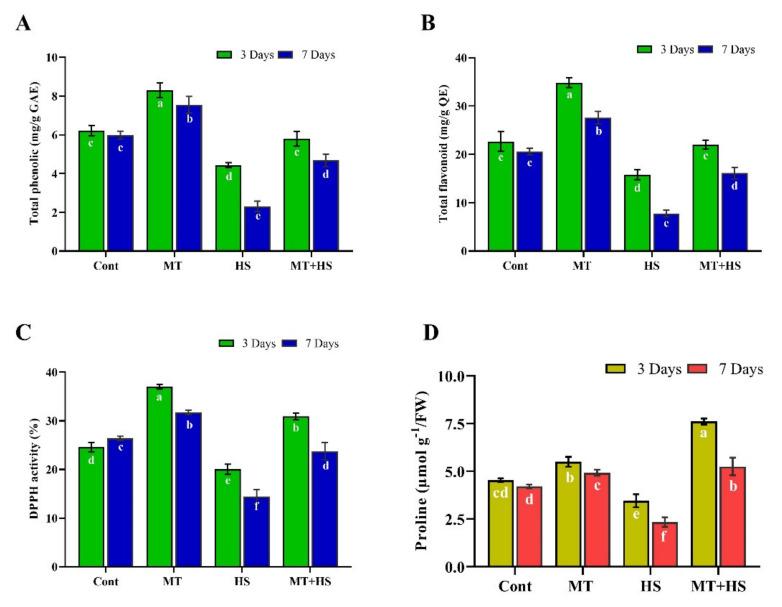
Effects of melatonin application with or without high temperature (42 °C) on (**A**) total phenolic content, (**B**) total flavonoid content, (**C**) DPPH activity, and (**D**) proline accumulation in soybean seedling after 3 and 7 days. Each data point is the mean of three replicates. Error bars represent the standard error of the mean. Bars with different letters are significantly different from each other by Duncan’s multiple range test at *p* ≤ 0.05.

The phenolic and flavonoid compounds are the most important antioxidants in plants and display ROS scavenging activities under stress conditions. As shown in (Figure 4), TPC, TFC, and antioxidant (DPPH) activity initially increased slightly in the MT-treated plants (without stress) but were significantly decreased by 28.6% and 61.5%, 30.2% and 58.8%, and 18.5% and 45.3% on day 3 and 7, respectively, compared to control plants (non-treated). Under heat stress, however, MT treatment caused a significant increase in the TPC (31.1%), TFC (39.0%), and (DPPH) activity (54.2%) by day 3, and a further improvement of 90.5%, 73.1%, and 54.3%, respectively, compared to the control plants (heat without MT treatment) (Figure 4A–C).

### 2.5. Effect of Exogenous MT on Proline Content in Soybean Plants under Heat Stress

Proline accumulation is a widespread response in plants to environmental stresses. It is a compatible osmolyte that can stabilize proteins and cells from the damaging effects of osmotic stress by decreasing the cytoplasmic osmotic potential, allowing osmotic adjustment and water retention to prevent dehydration. In the current study, the MT-treated plants showed only a slight difference in proline content from the control plants (no MT treatment) under the normal condition (no heat stress). Heat stress decreased the proline contents considerably by 23.7% on day 3 and 45.2% on day 7 compared to control plants (without stress) but increased by 64.5% and 86.4% in MT-treated plants compared to heat-stressed control plants (Figure 4D).

### 2.6. Exogenous MT Induces Endogenous MT under Heat Stress

**Figure 5:**
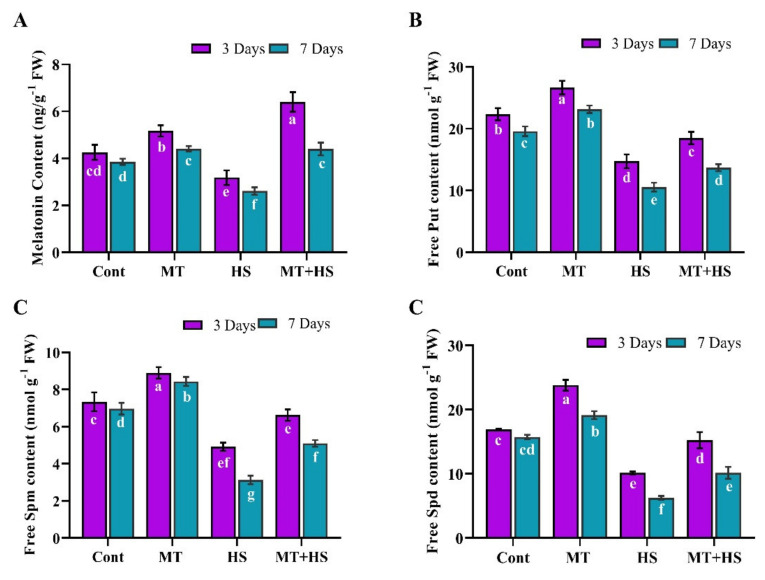
Effects of melatonin application with or without high temperature (42 °C) on (**A**) endogenous melatonin content, (**B**) polyamine putrescine, (**C**) spermine, and (**D**) spermidine in soybean seedling after 3 and 7 days. Each data point is the mean of three replicates. Error bars represent the standard error of the mean. Bars with different letters are significantly different from each other by Duncan’s multiple range test at *p* ≤ 0.05.

We also quantified the endogenous MT accumulation in soybean plants with or without heat stress. Under the normal condition (no stress), endogenous MT was significantly increased in the MT-treated plants compared to control plants at 3 days, then gradually decreased by day 7. After heat stress for 3 days, the endogenous MT content was significantly reduced by 26.6% compared to control plants (no stress), and the MT-treated plants increased the endogenous MT level compared to heat-stressed plants. Heat stress caused a further decrease of 36.8% by day 7, but this effect was alleviated in MT-treated plants, which exhibited a 56.7% increase in the proline content compared to the heat-stressed control plants without MT treatment (Figure 5A).

### 2.7. Effects of Exogenous MT on Endogenous PA Accumulation under Heat Stress

PAs are low molecular weight nitrogenous compounds integral to stress responses in plants. In the current study, we evaluated the effects of exogenous MT on the PA (Put, Spm, and Spd) accumulation during heat stress. After 3 and 7 days of heat stress, the soybean plants displayed a decrease in Put (40.0% and 52.7%), Spm (39.9% and 60.1%), and Spd (32.9% and 62.5%) compared to control plants (no stress). However, during the same time periods, Put (43.4% and 35.9%), Spm (50.1% and 61.7%), and Spd (34.7% and 68.5%) were significantly increased in MT-treated plants compared to their counterparts without MT treatment (heat stressed control plants) (Figure 5B–D).

### 2.8. Effects of Exogenous MT on Endogenous ABA and SA Biosynthesis during Heat Stress

**Figure 6:**
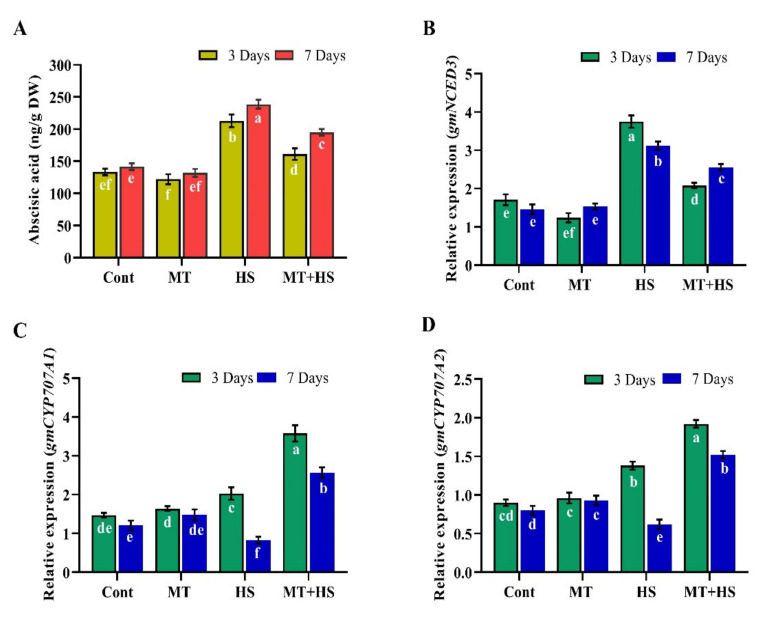
Effects of melatonin application with or without high temperature (42 °C) on (**A**) abscisic acid content, (**B**) relative expression of ABA biosynthesis gene gmNCED3, (**C**) relative expression of ABA catabolic gene gmCYP707A1, and (**D**) gmCYP707A2 in soybean seedling after 3 and 7 days. Each data point is the mean of three replicates. Error bars represent the standard error of the mean. Bars with different letters are significantly different from each other by Duncan’s multiple range test at *p* ≤ 0.05.

We investigated the possible influence of exogenous MT on ABA and SA biosynthesis in soybean plants during exposure to high-temperature stress. The results showed that after 3 and 7 days under the normal condition (no stress), MT did not affect the ABA and SA levels. Heat stress for 3 and 7 days significantly increased the endogenous ABA level by 39.5% and 58.6% compared to control plants (no heat stress). However, MT treatment significantly decreased the ABA level by up to 29.6% on day 3 of heat stress. The efficacy of endogenous MT was less apparent by day 7. In response, the ABA level in MT-treated plants was slightly increased by the stress condition but still significantly decreased by 18.2% compared to heat-stressed control plants without MT treatment (Figure 6A). To further confirm these results, we assessed the mRNA expression of key genes involved in ABA biosynthesis (NCED) and catabolism (CYP707A1 and CYP707A2). The results showed that heat stress enhanced the relative expression of NCED3 (76.9% and 29.4%) and decreased that of CYP707A1 (37.2% and 56.8%) and CYP707A2 (28.1% and 59.2%) compared to MT-treated plants after heat stress for 3 and 7 days. Meanwhile, significant down-regulation of NCED3 (44.5% and 19.2%) and significant up-regulation of CYP707A1 (59.6% and 109.1%) and CYP707A2 (39.1% and 86.4%) occurred in the plants pretreated with MT compared to control plants (without MT treatment) exposed to high-temperature stress for 3 and 7 days (Figure 6B–D).

**Figure 7:**
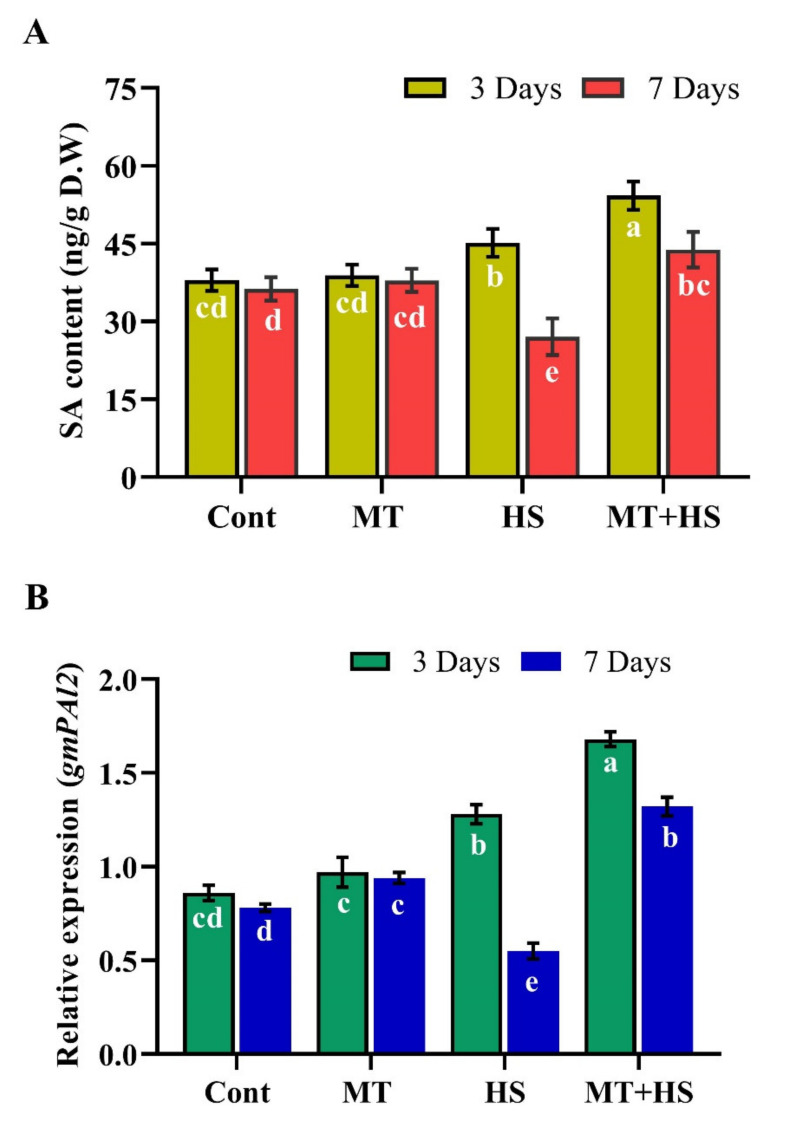
Effects of melatonin application with or without high temperature (42 °C) on (**A**) salicylic acid (SA) and (**B**) relative expression of SA biosynthesis gene gmPAL in soybean seedling after 3 and 7 days. Each data point is the mean of three replicates. Error bars represent the standard error of the mean. Bars with different letters are significantly different from each other by Duncan’s multiple range test at *p* ≤ 0.05.

Moreover, exogenous MT enhanced SA accumulation during heat stress. Under the normal condition (without heat stress), MT pretreatment did not affect the SA levels at 3 and 7 days **(Figure 7**A). However, SA increased slightly by 14.9% on day 3 in response to heat stress (42 °C) and decreased significantly by 31.6% on day 7 compared to the control plants (not exposed to heat stress), but accumulated to 24.6% (day 3) and 51.3% (day 7) in MT-treated plants during the heat exposure. A similar trend of the SA biosynthesis gene PAL2 was observed, in that MT pre-treatment caused significant up-regulation of PAL2 by 31.2% and 84.9% during heat stress for 3 and 7 days, respectively (Figure 7B).

### 2.9. Exogenous MT Regulates Relative Expression of HSPs

**Figure 8:**
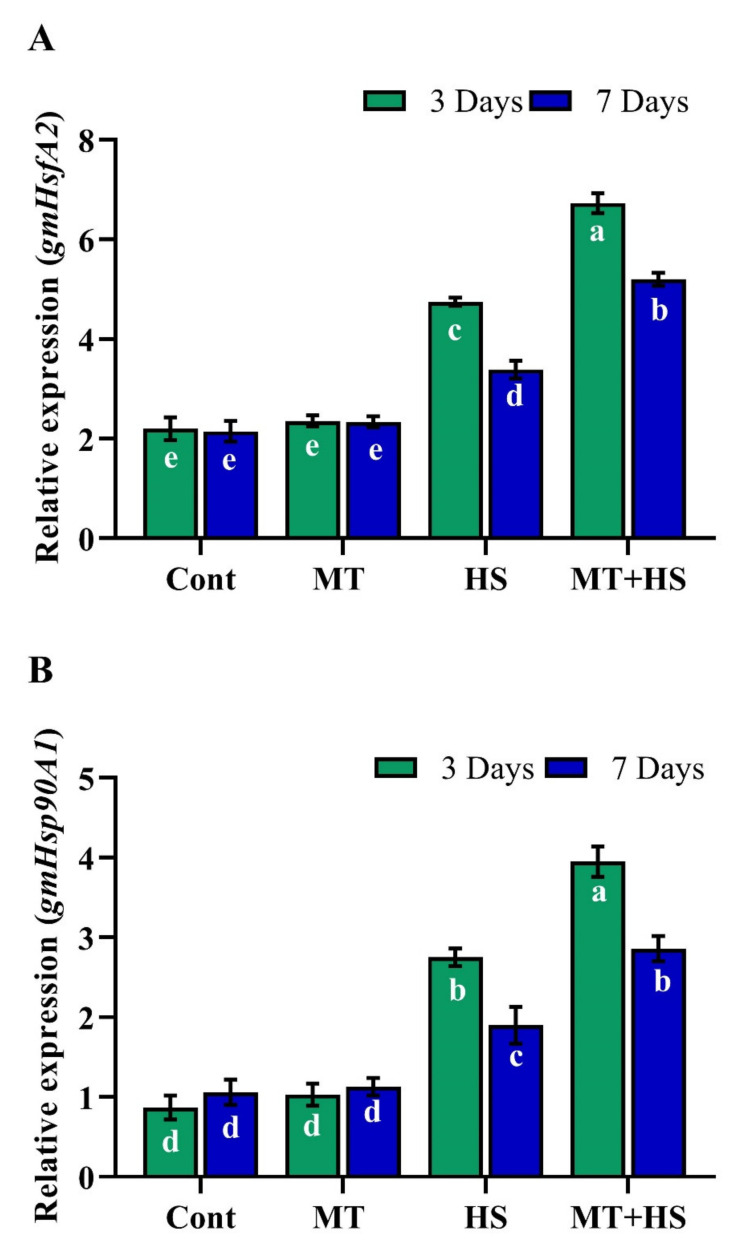
Effects of melatonin application with or without high temperature (42 °C) on (**A**) relative expression of heat response transcript factor gmHsfA2 and (**B**) relative expression of heat shock protein gmHsp90 in soybean seedling after 3 and 7 days. Each data point is the mean of three replicates. Error bars represent the standard error of the mean. Bars with different letters are significantly different from each other by Duncan’s multiple range test at *p* ≤ 0.05.

Under the normal condition (no heat stress), exogenous MT did not affect the expressions of gmHsfA2 and gmHsp90 (Figure 8A,B). After heat stress (42 °C) for 3 days, plants without MT treatment showed downregulated expression of gmHsfA2 (29.4% and 34.8%) and gmHsp90 (30.3% and 38.7%) compared to their MT-treated counterparts, which revealed upregulated expression of gmHsfA2 and gmHsp90 by 48.2% and 43.6%, respectively. Compared to heat stress for 3 days, after 7 days, the relative expression of gmHsfA2 and gmHsp90 decreased slightly in the control plants (no MT treatment, but remained upregulated by 59.6% and 50.3% in the MT-treated plants.

## 3. Discussion

The current study investigated the influence of exogenous MT and its role in soybean plants during high-temperature exposure. The results demonstrated a heat stress-induced reduction in plant growth and development coupled with a decrease in the SL, RL, biomass, and Chl content; however, exogenous MT counteracted these adverse effects of heat stress (Table 1). Similarly, Antoniou et al. [35] found that MT pretreatment improved Arabidopsis tolerance to prolonged drought stress. Moreover, under oxidative stress, MT application increased plant height and biomass [36].

Chl is involved in photosynthesis and plays an essential role in transmitting and absorbing light energy [37]. The current study showed that MT application significantly enhanced the biosynthesis of Chl a, Chl b, and carotenoids in soybean plants. Likewise, exogenous MT improved the growth and development of rice seedlings and enhanced the rate of photosynthesis and photosystem II activity by stimulating the antioxidant enzymes to alleviate the accumulation of ROS and MDA due to cell death induced by cold stress [38].

Under biotic and abiotic stress conditions, plants generate ROS, such as H_2_O_2_ and O_2_^−^, which accumulate in the cell, causing oxidative damage, including peroxidation of membrane lipids and increased MDA and electrolyte leakage contents [5]. Similar to our previous finding Imran et al. 2021 [39] observed decreased MDA and electrolyte leakage levels and scavenging of excess H_2_O_2_ induced by drought stress in MT-pretreated soybean plants. In the current study, heat-stress in soybean plants leads to increased production of stress indicators, including H_2_O_2_ and O_2_^−^, and subsequent elevation of the MDA and electrolyte leakage contents, which were counteracted by pretreatment with MT (Figure 1). Similar effects of MT pretreatment on ROS scavenging reported in tomato [5,40] and kiwifruit [20] under heat stress have been largely attributed to improved redox status coupled with enhanced activities of antioxidant enzymes. Furthermore, to protect plants from oxidative damage and scavenge the ROS, plants activate several antioxidant enzymes, such as POD, SOD, CAT, APX, and GSH, that are involved in the antioxidant defense system and serve as key regulators to inhibit ROS accumulation [41].

In this context, we also determined the endogenous levels of POD, SOD, CAT, APX, and GSH in soybean plants under heat stress following pretreatment with MT. The heat stress-induced decrease in the antioxidant enzymes (POD, SOD, CAT, APX) as well the non-enzymatic antioxidant [7] levels and increased MDA and electrolyte leakage contents were reversed by MT application, as confirmed by the up-regulation of antioxidant-related genes, such as *GmPOD1*, *GmSOD, GmCAT1*, and *GmAPX*, whose expression levels were decreased by heat stress (Figure 2). This ability of MT to improve the detoxification of ROS and enhance the plant tolerance to stress conditions may be associated with the role of MT as an antioxidant that neutralizes various free radicals, ROS, and reactive nitrogen species while stimulating antioxidant enzymes, such as CAT and POD, which convert H_2_O_2_ into H_2_O and O_2_ [42,43]. To further investigate the antioxidant substances, we measured the TPC and TFC levels and the DPPH free radical scavenging activity (Figure 4). Accordingly, heat stress caused a significant reduction in the antioxidant substances and antioxidant capacity, which, conversely, were enhanced by pretreatment with MT. Similar results were reported in kiwifruit seedlings [44] and basil plants [45] under salinity stress. In our experiments, pretreatment with 100 µmol MT also increased the endogenous MT level during heat stress (Figure 5), consistent with Xu et al. [25] and Jahan et al. [46] in cucumber and tomato plants, respectively, during heat stress, and this could have contributed to augmenting the antioxidant defense mechanisms. The present study revealed a significant decrease in the PAs (Put, Spd, and Spm) due to heat stress. By contrast, exogenous MT increased the level of free PAs (Put, Spd, and Spm) compared to control plants (heat-treated) (Figure 5). Exogenous MT has already been shown to increase PA regulation and enhance tolerance to heat stress in tomato plants and alkaline stress in *M. hupehensis* plants [5,47]. Shi and Chan [48] reviewed and supported the putative connections between polyamine metabolism fluxes and plant tolerance to stresses, such as salinity, high temperature, and drought.

ABA and SA are plant hormones with important roles in plant abiotic stress responses, growth, and development and are considered the first line of defense against abiotic and biotic stresses. The production of ABA in plants cell is associated with ROS formation [49], and ABA accumulation may lead to an increase in H_2_O_2_ accumulation [50]. Exogenous MT has been shown to decrease the ABA content and downregulate its biosynthesis genes, such as NCED while upregulating its catabolic genes CYP707A1 and CYP707A2 [51]. In support of those findings, the present results revealed an increase in the accumulation of both ABA and the key ABA biosynthesis gene *GmNCED3* upon exposure to high temperature, effects mitigated by endogenous MT, which inhibited the over-accumulation of ABA, downregulated *NCED3*, and upregulated *CYP707A1* and *CYP707A2* relative expression levels, further promoting heat tolerance in soybean plants (Figure 6). These results also confirm the previous finding of Zhang et al. [30] that MT application decreases the ABA content and *NCED* and *bZEP* expression and increases the cytokinin biosynthesis in perennial ryegrass under heat stress. Similar results were also demonstrated in Malus species under drought stress [51] and Pinellia ternate under heat stress [52]. Similarly, the present results showed that in the plants exposed to 42 °C, the SA content increased during the first 3 days, then gradually decreased as the stress condition was prolonged. However, MT application enhanced the SA content and increased the expression of the SA biosynthesis gene *PAL2* during heat stress (Figure 7). Similarly, in pathogen-susceptible Arabidopsis serotonin N-acetyltransferase (SNAT) knockout mutants, exogenous MT treatment increased the SA levels and restored the induction of defense gene expression, eliciting pathogen resistance [53]. Previously, MT treatment increased SA and nitric oxide accumulation in tobacco plants in response to viral infection [54]. Intriguingly, these regulatory molecules have so far been studied separately, and although they share a common biosynthetic precursor, chorismic acid, which is generated from shikimic acid, and have similar stress regulation signals and physiological functions [55]. The complex relationship between MT and SA crosstalk should be further investigated at the transcriptomic level as well as knock-out, knock-down, and overexpressing MT and SA biosynthesis genes in order to widen their use in future breeding programs of stress tolerance. Furthermore, melatonin acts through receptor-dependent and -independent mechanisms, in a context-dependent manner in a cell that melatonin can rapidly produce and metabolize through cellular components include malignant cells. Similarly, melatonin metabolism can occur through three pathways including kynuric, indolic, and CYP-mediated pathways with metabolites by 6-hydroxymelatonin (AFMK) and (AMK) in human skin. Moreover, 6-hydroxymelatonin, AFMK, and 4-hydroxymelatonin can be produced in the epidermis through UVB-induced non-enzymatic melatonin. These metabolites are similar to lower organisms and plants which indicate the phylogenetic conservation among diverse species and adaptation to the primordial defense mechanism. Likewise, melatonin and its metabolites counteract environmental stress to maintain homeostasis through broad-spectrum activities, regulating both the degradation and melatoninergic pathways, since the phenotypic regulations mostly depend on melatonin concentration and its metabolites [13,56].

The HSFs and HSPs are the most important regulators in maintaining protein structure and play a vital role in ROS detoxification through the H_2_O_2_ -mediated signaling pathway [5,57]. In the present study, we also examined the effects of heat stress and MT-pretreatment on gmHsfA2 and gmHsp90 in soybean seedlings. MT-pretreatment led to a significant up-regulation in the expression of *gmHsfA2* and *gmHsp90* during heat stress compared with the respective control (heat-stressed but no MT pretreatment) (Figure 8). Similar results were reported in tomato seedlings exposed to high-temperature conditions [18]. Similarly, melatonin and its derivatives have a potential role in protecting UVB-induced oxidative stress and DNA damage, which results to improve the cellular, genomic, and tissue integrity against UVB-induced [27]. Melatonin also shows a positive impact on the reduction of heat shock Hsp70 protein in UVR-treated of full-thickness skin in organ culture and cultured keratinocytes [28].

Such observations may indicate that exogenous MT mitigates heat stress-induced oxidative damage by activating *GmHsfA2* and *GmHsp90*. Recent reports also revealed that *HsfA2* plays a positive role to maintain H_2_O_2_ signaling and increases heat stress resistance, and *HSP90* coordinates to enhance the DNA-binding process in plants exposed to heat stress, and this whole mechanism might be related to MT-mediated heat tolerance [26,58].

## 4. Materials and Methods

### 4.1. Plant Material and Growth Condition

Soybean seeds were provided by the Soybean Genetic Resource Centre, Kyungpook National University, Daegu, Korea. The seeds were first surface-sterilized with 2.5% sodium hypochlorite for 5 min, washed three times with distilled water, and then left to germinate in germination trays filled with horticultural soil containing peat moss (10–15%), coco peat (45–50%), perlite (35–40%), zeolite (6–8%) with NO3 (ca. 0.205 mg/g), NH+ (ca. 0.09 mg/g), KO (ca. 0.1 mg/g), and PO (ca. 0.35 mg/g) [59], in a growth chamber at 24–26 °C, 55–65% relative humidity, 14/10 h day/night, and light intensity of 1000 µEm2/s from sodium lamps. When the unifoliate leaves fully emerged, the uniformly germinated seedlings were selected and transferred to a plastic pot filled with the same horticulture substrate as mentioned above. All the plants were grown in a growth chamber under the same condition used for germination. Five days after transplanting into pots, plants were first pretreated with 30 mL of 100 µmol MT twice daily for 6 days to the root zone [60]. At the V2 stage (when second trifoliate leaves start developing), plants were exposed to heat stress of 42 °C for 3 and 7 days. Four different treatments were studied: (1) control plants (distilled water), (2) 100 µmol MT treatment, (3) high-temperature stress (42 °C), (4) high-temperature (42 °C) + 100 µmol MT treatment. Control plants were kept in a separate growth chamber at 24–26 °C, 14/10 h day/night. After completing the stress period, the chlorophyll content of plants was measured using a SPAD-502 Chl meter (Konica Minolta, Japan). The plants were subsequently harvested. Root length (RL) and shoot length (SL) were measured using a scale, then the plants were snap-frozen in liquid N2 and stored at −80 °C until further analysis.

### 4.2. Determination of Antioxidant Enzymatic Activity

CAT activity was determined by calculating the H_2_O_2_ absorption reduction at 240 nm, as previously described [61]. The reaction buffer contained 50 mM potassium phosphate buffer (pH 7.8) and 15 mM H_2_O_2_. Then, 100 μL of the enzyme extract was added to the reaction mixture to initiate the reaction. CAT activity was assessed by measuring the H_2_O_2_ level in the reaction mixture after 1 min using the extinction coefficient (ε) of 40 mM/cm. POD activity was assayed by the guaiacol method [62], performed by adding 0.1 mL of the supernatant to the reaction mixture containing 1.0 mL of 2% H_2_O_2_, 2.9 mL of 50 mM phosphate buffer (pH 5.5), and 1.0 mL of 50 mM guaiacol. Phosphate buffer was used as the control without enzyme. The absorbance was read at 470 nm for 3 min, and POD activity was calculated as the unit change per minute. SOD activity was measured as previously described [60,63] by evaluating its inhibitory effect on the photochemical reduction of nitro blue tetrazolium (NBT). SOD activity units were determined as the amount of enzyme required to cause 50% inhibition of the reduction of NBT, as monitored at 560 nm. For measuring the APX activity, 100 mg of the plant sample was extracted with 1 mL of 50 mM phosphate buffer (pH 7.0) containing 1 mM ascorbic acid and 1 mM EDTA. The homogenate was centrifuged at 4830× *g*, 4 °C for 15 min. The supernatant was mixed with phosphate buffer solution (pH 7.0), 15 mM ascorbic acid, and 0.3 mM H_2_O_2_, and the reaction mixture was read at 290 nm. To determine the GSH content, a previously detailed method [60] was used.

### 4.3. Determination of O_2_^−^ and MDA, and Histochemical Detection of H_2_O_2_

For O_2_^−^ determination, the method of Jahan et al. [5] was followed. Briefly, 0.2 g of fresh leaves were homogenized with 2 mL of phosphate buffer (50 mM, pH 7.8), followed by centrifugation at 10,000× *g*, 4 °C for 15 min. Then, 0.1 mL of 10 mM hydroxylamine hydrochloride and 0.5 mL of phosphate buffer (50 mM, pH 7.8) were mixed with 0.5 mL of the supernatant, followed by incubation at room temperature (RT) for 25 min. After incubation, 1 mL of 7 mM naphthylamine and 17 mM sulfanilamide were added to the mixture and further incubated at RT for 30 min. The absorbance was read at 530 nm, and the O_2•_^−^ production was calculated with a standard curve of NaNO_2_ (expressed as nmol g/min/fresh weight [FW]). Lipid peroxidation in leaves was determined by measuring the MDA levels (μmol/g FW), as described elsewhere [39]. Briefly, 0.1 g of fresh plant tissue was ground with 10 mL of 5% trichloroacetic acid (TCA) and centrifuged at 6000× *g*, 4 °C for 10 min. The resulting supernatant was suspended in 4 mL of thiobarbituric acid, heated at 90 °C for 25 min, and immediately cooled down at 4 °C. The sample was centrifuged, and the supernatant was read at wavelengths of 532 and 600 nm.

The H_2_O_2_ content was measured using a previously described method [39,64]. Briefly, 0.2 g of leaf sample was ground and extracted using 5 mL of 0.1% TCA, then centrifuged at 12,000× *g* for 15 min. Next, 0.5 mL of the supernatant was collected, and 1 mL of 1 M KI and 0.5 mL of 10 mM phosphate buffer (pH 7.0) were added, and the absorbance was detected at 390 nm. The H_2_O_2_ content (expressed as μM/g dry weight [DW]) was estimated using ε = 0.28 mM/cm. For histochemical detection of H_2_O_2_, the leaves were kept in a vacuum with 0.5 mg/mL of DAB solution, prepared by dissolving the DAB in 25 mM Tris-HCl (pH 3.8). After incubation at RT for 12 h, brown spots appeared on the surface of leaves because of the reaction between DAB and H_2_O_2_. The leaves were subsequently bleached with 95% ethanol and incubated at 85 °C in a water bath for 30 min to remove Chl.

### 4.4. Determination of Electrolyte Leakage and Proline Content

Electrolyte leakage was determined using a (Huriba B-173 Twin Cond electrical conductivity meter, Minami-Ku, Kyoto, Japan), as described previously [38]. Proline content was measured as detailed elsewhere [39,65]. Briefly, 0.3 g of fresh plant sample was homogenized with 5 mL of sulfosalicylic acid and mixed by vortex for 1 min, then homogenized with 2 mL of ninhydrin reagent and glacial acetic acid and heated at 80 °C in a water bath for 30 min. After cooling at RT, the reaction solution was centrifuged at 10,000× *g* for 10 min. The supernatant was read at 520 nm, and the proline content was calculated according to the standard curve.

### 4.5. Determination of Chl a, Chl b, and Carotenoid Contents

Chl a, Chl b, and carotenoids were quantified using the previously described methods [66]. Briefly, 0.5 g of fresh plant sample was homogenized with 80% acetone, followed by vortex for 2 min and incubation at RT for 30 min. The homogenate was centrifuged at 11,000× *g* for 10 min, and the supernatant was read at 470, 645, and 663 nm, respectively. Chl a, Chl b, and carotenoid contents were calculated as follows:

Chl a (mg/g FW) = [(12.7 × A663) − (2.69 × A645)/100 × W] × V(1)

Chl b (mg/g FW) = [(22.9 × A645) − (4.68 × A663)/100 × W] × V(2)

Carotenoids (µg/g FW) = A480 + (0.638 × A663) − (0.638 × A645)(3)

### 4.6. Determination of Phenolic Compounds, Flavonoids, and DPPH Activity

Total phenolic content (TPC) and total flavonoid content (TFC) were quantified using the method of Liang et al. [20]. Briefly, 0.2 g of fresh plant sample was homogenized with 70% methanol containing 2% of formic acid and 28% ethanol, followed by ultrasonication for 30 min and centrifugation at 10,000× *g* for 10 min. The supernatant was filtered through a 0.45-µm membrane filter. TPC was quantified by the Folin-Ciocalteu method using gallic acid as the standard, and the absorbance was read at 765 nm. To determine the flavonoid content, the absorbance was read at 510 nm and expressed as rutin equivalents. DPPH scavenging activity was determined as a detailed method of [67].

### 4.7. Extraction and Quantification of Endogenous MT

Endogenous MT was extracted and quantified in soybean plant leaves using the Melatonin ELISA Kit (Enzo Life Sciences, Farmingdale, NY, USA) according to the manufacturer’s protocol [35,60]. In brief, the soybean leaves were rinsed thrice with distilled water and wiped with a paper towel. Once cleaned, 0.1 g of leaves were ground to a fine powder using liquid N_2_ and homogenized in 125 µL of 1X stabilizer, followed by the addition of 750 µL of cold ethyl acetate and vortexed. After the mixture was incubated on ice for 5 min, it was centrifuged at 5000× *g* for 10 min. The organic layer was transferred to a fresh tube and dried under N_2_ gas. The pellet was suspended in 250 µL of 1X stabilizer for further quantification according to the manufacturer’s protocol.

### 4.8. Quantification of PAs

Endogenous free PA content was analyzed as previously described by Jahan et al. [5]. Briefly, 0.3 g of fresh leaf tissue was homogenized in 5% cold perchloric acid, followed by incubation on ice for 1 h. The homogenate was centrifuged at 12,000× *g* for 20 min, and the upper supernatant was used to determine the free PAs. After that, a 0.7-mL aliquot was reacted with 1400 µL of 2N NaOH with 15 μL of benzoyl chloride. After a gentle vortex, it was incubated at 37 °C for 30 min. Two milliliters of saturated NaCl were added to the solution to stop the reaction. To extract benzoyl PAs, 2 mL of cold diethyl ether was mixed into the solution and centrifuged at 3000× *g* for 5 min. The extract was evaporated to dryness, then resuspended in 1 mL of 64% methanol. For chromatographic separation and quantification of the PAs (putrescine [Put], spermine [Spm], spermidine [Spd]), we used a UHPLC instrument (Ultimate 3000, Thermo Scientific, San Jose, CA, USA) equipped with a C18 reversed-phase column and a flow rate of 0.8 mL/min.

### 4.9. Extraction and Quantification of the Phytohormones ABA and Salicylic Acid (SA)

Endogenous ABA was quantified and extracted as described previously [68], with slight modifications reported in a recent study [69]. ABA was extracted from the aerial parts of the plant (freeze-dried plant samples, 0.3 g), and a chromatograph was run using the Me-[2H6]-ABA standard. The fraction was methylated with diazomethane for subsequent detection and quantification of ABA by GC-MS (6890N Network GC System, Agilent Technologies). The software from ThermoQuest Corp. (Manchester, UK) was used to monitor signal ions (*m*/*z* 162 and 190 for Me-ABA, and *m*/*z* 166 and 194 for Me-[2H6]-ABA) (Table S1).

Endogenous SA was extracted and quantified using previously described methods [39,70]. Briefly, 0.3 g of the freeze-dried plant sample was treated with 5 mL of 100% methanol, followed by centrifugation at 10,000× *g* (three times). The pooled methanolic extracts were vacuum dried. The dried pellets were suspended in 2.5 mL of 5% TCA and centrifuged at 10,000× *g* to collect the supernatant, which was separated using ethyl acetate, cyclopentane, and isopropanol (ratio of 100:99:1, *v*/*v*/*v*) and dried using N_2_ gas, followed by quantification by HPLC-fluorescence detection (Shimadzu RF-10AXL, Kyoto, Japan) (Table S2).

### 4.10. RNA Extraction and Quantitative Real-Time PCR Analysis

RNA was extracted using the method previously described by Khan et al. [71] with slight modifications. Briefly, 0.1 g of fresh leaf sample was ground in liquid N_2_, then immediately transferred to an RNase-free E-tube containing extraction buffer: 0.05 M Tris-HCl, pH 7.5; 0.25 M NaCl; 20 mM EDTA 8; 4% (*w*/*v*) PVP; and 1% (*w*/*v*) SDS [72]. The RNA quality and concentration were measured using (Optizen NANO Q, Mecasys Co., Ltd. Daejeon, Korea). Then, cDNA synthesis and quantitative real-time PCR (qRT-PCR) were performed as described by Imran et al. [73]. Briefly, about 1 µg of RNA was used to synthesize cDNA by using the BioFACT™ RT kit (BioFACT™, Daejeon, Korea), according to the manufacturer’s instructions. The synthesized cDNA was used as the template for further evaluation of transcript accumulation using qRT-PCR (Eco™ Illumina™, California, CA, USA). The detailed list of genes and their corresponding primers are shown in Table S3. A 2X real-time PCR Master Mix (BioFACT™), along with 10 µM of each gene-specific primer and 100 ng of template cDNA in a final reaction volume of 20 µL, were used. A two-step PCR was performed for 40 cycles under the following conditions: polymerase activation at 95 °C for 15 min, denaturation at 95 °C for 15 s, annealing and extension at 60 °C for 30 s. A “no template control” was used as the negative control. The expression of each gene was compared to actin as an internal control, and the experiment was repeated in triplicate.

## 5. Conclusions

Based on our findings, we have described a probable mechanism by which MT mitigates the adverse effects of heat stress in soybean seedlings. We observed that exogenous MT application enhanced the heat tolerance of soybean plants through scavenging the excess ROS (O_2_^−^, H_2_O_2_) and reduced the MDA and electrolyte leakage contents by activating the antioxidant enzyme activities, up-regulating antioxidant-related gene expression, and increasing the proline accumulation. In addition, MT treatment elevated the endogenous PA and SA contents, up-regulated the SA biosynthesis gene PAL2, reduced the endogenous ABA level, downregulated the ABA biosynthesis gene NCED3, and upregulated two ABA catabolic genes CYP707A1 and CYP707A2. Therefore, we concluded that MT suppressed heat stress-induced oxidative damage, which may coordinate with the PAs and SA biosynthesis pathways, to detoxify the over-accumulation of ROS. These findings provide novel insight into the crosstalk among MT, phytohormones, and PAs to inhibit the effects of heat stress. To understand these interactions, further investigation is required to determine how these molecules collectively collaborate to alleviate heat stress-induced oxidative damage.

## Figures and Tables

**Table 1:** Effects of melatonin application on growth attributes of soybean plants 3 and 7 days after heat stress.

Treatment	SL	RL	FW	DW	CHL (SPAD)
3 Days					
Control	15.7 ± 0.3 b	14.4 ± 0.3 b	2.1 ± 0.1 b	1.8 ± 0.1 b	37.3 ± 0.2 b
MT	17.2 ± 0.5 a	16.7 ± 0.6 a	2.8 ± 0.1 a	2.1 ± 0.06 a	39.8 ± 0.2 a
HS	13.1 ± 0.6 c	13.3 ± 0.5 c	1.4 ± 0.06 c	1.02 ± 0.1 d	34.4 ± 0.6 c
MT/SH	15.2 ± 0.2 b	14.1 ± 0.4 b	2.0 ± 0.09 b	1.5 ± 0.1 c	37.1 ± 0.2 b
7 Days					
Control	18.2 ± 0.2 ab	17.6 ± 0.2 b	2.9 ± 0.06 b	2.2 ± 0.2 b	39.3 ± 0.5 b
MT	19.8 ± 0.5 a	21.7 ± 0.6 a	3.4 ± 0.1 a	2.7 ± 0.05 a	41.7 ± 0.9 a
HS	14.4 ± 0.2 c	14.1 ± 0.3 c	1.7 ± 0.09 d	1.1 ± 0.03 d	28.9 ± 0.8 d
MT/SH	16.8 ± 0.2 b	16.9 ± 0.3 b	2.2 ± 0.08 c	1.7 ± 0.08 c	34.8 ± 0.5 c

The treatment includes control (without any treatment or stress), MT (Melatonin treated), HS (High temperature 42 °C), MT/HS (Melatonin + High temperature 42 °C), while the measurement includes SL (Shoot length), RL (Root length), FW (Fresh weight), DW (Dry weight) and Chl (Chlorophyll content). Each data point is the mean of at least three replicates. The mean (± SE) followed by the different letter (s) are significantly different from each other as evaluated by DMRT.

## Data Availability

Not applicable.

## Supplementary Materials

The following are available online, Table S1: the GC/MS-SIM condition for the quantification of abscisic acid. Table S2: HPLC condition used for analysis and quantification of salicylic acid. Table S3: List of primers sequences used for real-time PCR analysis. Figure S1: Effects of melatonin (MT) application on (A) Chlorophyll a, (B) Chlorophyll b and (C) carotenoids. With or without high temperature stress in soybean plants. Each data point is the mean of three replicates. Error bars represent standard error of mean. Bars with different letters are significantly different from each other as evaluated by DMRT at *p* ≤ 0.05.

## Sample Availability

The datasheet used or analyzed samples of the compounds and plants material are available from the authors.
