# Supplementary material for: Melatonin Ameliorates Thermotolerance in Soybean Seedling through Balancing Redox Homeostasis and Modulating Antioxidant Defense, Phytohormones and Polyamines Biosynthesis
Source: Molecules. 2021 Aug 24;26(17):5116. doi: 10.3390/molecules26175116 (PMC8434054; doi:10.3390/molecules26175116)
Supplement: Supplementary file 1 [file molecules-26-05116-s001.zip › molecules-1328536-supplementary.pdf]

Research Article

Melatonin ameliorates thermotolerance in soybean seedling through balancing redox homeostasis and modulating antioxidant defense, phytohormones and polyamines biosynthesis

Muhammad Imran[1], Muhammad Aaqil Khan[1], Raheem Shazad[2], Saqib Bilal[3], Murtaza Khan[1], Byung-Wook Yun[1], Abdul Latif Khan[3]*, In-Jung Lee[1]*

1 School of Applied Biosciences, Kyungpook National University, 41566. Republic of Korea.
2 Department of Horticulture, University of Haripur, Haripur 22620, Pakistan.
3 Natural & Medical Sciences Research Center, University of Nizwa, 616. Sultanate of Oman
* Correspondence: ijlee@knu.ac.kr, Tel: + 82-53-950-5708
* Co-Correspondence: abdullatif@unizwa.edu.om, Tel: +968-2544-6358

Supplementary Table. S1: GC/MS-SIM condition for the quantification of abscisic acid

Equipment	Hewlett-Packard 6890, 5973N Mass Selective Detector
Column	HP-1 capillary column (30mx0.25mm i.d. 0.25µm film thickness) (J & W Scientific Co., Folsom, CA, USA)
Carrier gas	He (40 ml/min); head pressure of 30kpa
Source temperature	250℃
Oven condition	60℃ (1min) → 15℃/min →200℃ →5℃/min →250℃ →10℃/min →280℃
Injector temperature	200
Ionizing voltage	70 ev

Supplementary Table. S2: HPLC condition used for analysis and quantficantion of salicylic acid.

Equipment	Shimadzu LC-10
Column	HP hypersil ODS (particle size 5µm, pore size 120 A)
Wave length	Excitation 305 nm, Emission 365 nm
Detector	RF-10Axl (fluorescence detector)
Oven condition	60℃ (1min) → 15℃/min →200℃ →5℃/min →250℃ →10℃/min →280℃
Solvent A	100% MeOH
Solvent B	100% water in 0.5% acetic acid
Flow rate	1.0 ml/min

Supplementary Table. S3: List of primers sequences used for real-time PCR analysis

Gene	Forward	Reverse
SOD1{Cu-Zn}	5’- GGTACTGTCAGCTTCACTATTACT-3’	5’- ACCGCCCTTCCTATGATGTT-3’
CAT 1	5’- GGCAGCCCCGAAACCCTCAG-3’	5’- TGAGGCCATCGCGGACGAAG-3’
APX1	5’- CACTCTGCTGGAACCTTTGAC-3’	5’- AGAACCCTTAGTGGCATCGG-3’
POD	5’- TCTCTTCCCTTCAGAAGCTCAA-3’	5’- ATCAAAGCCCCGAACGGAAT-3’
HSP90A1	5’- CTCGTGAGATGCTGCAACAAA-3’	5’- TCTCCGCTCTTTGTCGAGTG-3’
HsfA-2	5’- ACTCGCGACAGTTTCATCGT-3’	5’- ACGGTCCGAATCAACCTTCC-3’
NCED3	5’- ACCACCTCTTCGACGGCGACGGAATGGT-3’	5’- ATGGCGAGGAGTTTTCCGTTGAAGAAGA-3’
CYP707A1	5’- CAGGCTCAATGGGGTGGCCG-3’	5’- AAAGCGTGCAGCCTCAGGGC-3’
CYP707A2	5’- GCCCCGAGGCTGCCAAGTTT-3’	5’- CACGTCAGCACACTGGCGGT-3’
PAL2.1	5’- GAGTACCGGAGGCCCGTCGT-3’	5’- CACCGAACCCGGTGGTGACG-3’
ACT11	5’- ATCTTGACTGAGCGTGGTTATTCC -3’	5’- GCTGGTCCTGGCTGTCTCC -3’

Supplementary Fig. S1

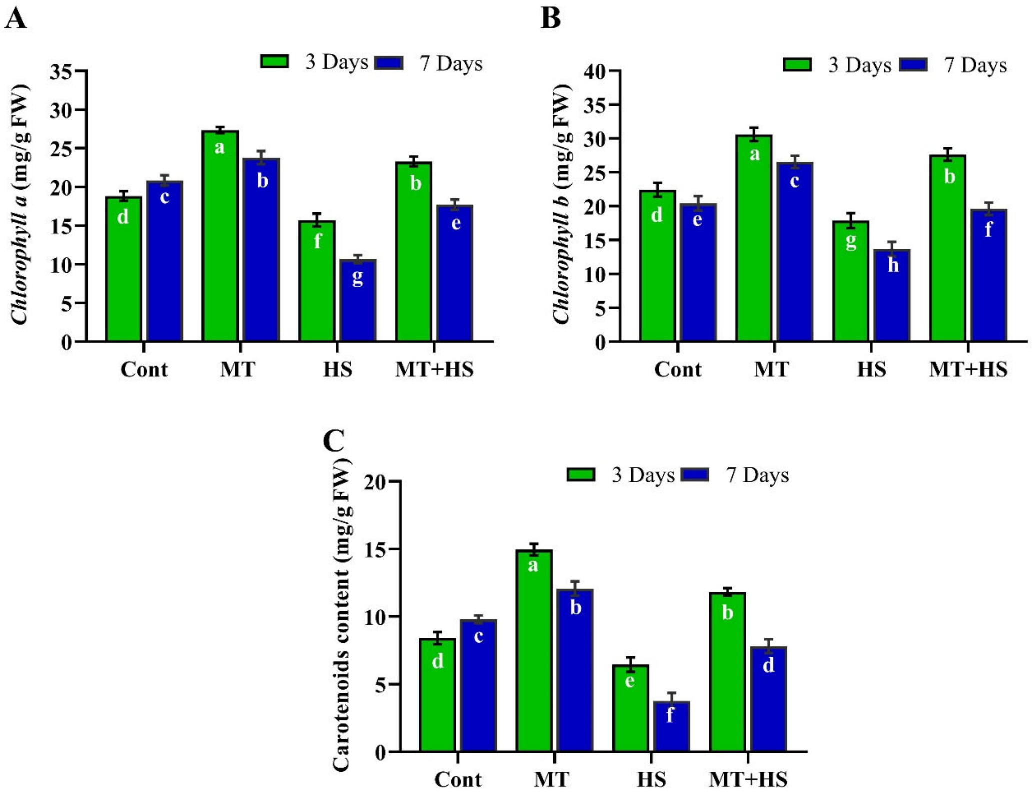

Supplementary Fig. 1 Effects of melatonin (MT) application on (A) *Chlorophyll a,* (B) *Chlorophyll b* and (C) carotenoids. With or without high temperature stress in soybean plants. Each data point is the mean of three replicates. Error bars represent standard error of mean. Bars with different letters are significantly different from each other as evaluated by DMRT at $p \leq 0.05$.
